# Cell death pathways in the pathogenesis of depression: mechanisms and therapeutic implications

**DOI:** 10.3389/fcell.2026.1966325

**Published:** 2026-09-21

**Authors:** Yuting Xiong, Yuxin Zhang, Hongxin Zhou, Gui Xiao

**Affiliations:** 1 Department of Nursing, Jiangxi University of Chinese Medicine, Nanchang, Jiangxi, China; 2 College of Integrated Traditional Chinese and Western Medicine, Jiangxi University of Chinese Medicine, Nanchang, Jiangxi, China

**Keywords:** apoptosis, depression, ferroptosis, necroptosis, neuroinflammation, pyroptosis, therapeutic targets

## Abstract

Depression is a highly prevalent and disabling psychiatric disorder with a complex and incompletely understood pathophysiology. Increasing evidence implicates regulated cell death (RCD), including apoptosis, necroptosis, pyroptosis, and ferroptosis, in neuronal vulnerability, synaptic impairment, and circuit dysfunction associated with depression. This review provides a comprehensive overview of the current understanding of distinct cell death modalities in animal models of depression and human brain tissues, with particular emphasis on their molecular mechanisms and pathological consequences. We further discuss the intricate interactions between cell death pathways and key pathological processes implicated in depression, including neuroinflammation, oxidative stress, mitochondrial dysfunction, and impaired neurotrophic support. In addition, we summarize emerging therapeutic strategies targeting cell death-associated signaling pathways, including pharmacological inhibition of specific cell death programs and modulation of shared upstream regulators. By integrating recent advances in molecular neuroscience and psychiatric research, this review provides a conceptual framework for elucidating the cellular mechanisms underlying depression and to highlight potential targets for the development of novel antidepressant therapies.

## 1 Introduction

Major depressive disorder (MDD), characterized by persistent low mood, anhedonia, and cognitive impairment (Marx et al., 2023). Although depression results from interactions among genetic, environmental, psychological, and biological factors, the monoamine hypothesis alone cannot explain its heterogeneous pathophysiology or the limited efficacy of current antidepressants (Peterson et al., 2018; Drevets et al., 2022). Increasing evidence implicates impaired neuroplasticity, mitochondrial dysfunction, neuroinflammation, and structural abnormalities in mood-regulating regions, particularly the hippocampus and prefrontal cortex (PFC) (Fernström et al., 2021; Chen Y. et al., 2024; Myoraku et al., 2022). Reduced gray matter volume in the hippocampus and other subcortical regions has been reported in patients with depression (Sacchet et al., 2015), whereas postmortem studies have identified increased caspase-8 expression and reduced cellular proliferation in the PFC (Miguel-Hidalgo et al., 2014), indicating persistent cellular stress and impaired tissue homeostasis.

Regulated cell death (RCD) has emerged as a potential link between chronic stress and neuronal dysfunction. Apoptosis, necroptosis, pyroptosis, and ferroptosis have been implicated in MDD through distinct but interconnected mechanisms (Zeb et al., 2022; Deng et al., 2022; Cao et al., 2021; Jia et al., 2026). Apoptotic alterations in MDD coexist with mitochondrial abnormalities and inflammatory activation (Scaini et al., 2022; Shelton et al., 2011). In stress-induced models, Zgasdermin D (GSDMD) signaling promotes pyroptosis and astrocytic loss, whereas dysregulation of FTO, BECN1, and iron metabolism is associated with ferroptotic vulnerability and depressive-like behaviors (Li et al., 2021a; Meng et al., 2025; Chen et al., 2023). These pathways share upstream drivers, including reactive oxygen species, inflammatory cytokines, mitochondrial dysfunction, and impaired autophagy, potentially forming a self-amplifying network of neuronal injury (Simon et al., 2021; Tang et al., 2021). Consistent with this framework, selective serotonin reuptake inhibitors modulate NLRP3-associated inflammation, while ginsenoside Rb1 and ketamine regulate ferroptosis through redox- and autophagy-related mechanisms (Alcocer-Gómez et al., 2017; Zhang et al., 2022). This review examines the molecular mechanisms, pathological roles, crosstalk, and therapeutic implications of major RCD pathways in depression (Table 1).

**TABLE 1:** Major regulated cell death pathways involved in depression.

Type	Molecular mechanisms	Major cell types/regions	Pathological effects	Potential targets
Apoptosis	BAX/BCL-2 imbalance; cytochrome c; caspase-9/3; Fas/TNFR1–caspase-8; BDNF–TrkB–PI3K/AKT suppression	Neurons; hippocampus, PFC	Neuronal loss, impaired neurogenesis and synaptic plasticity	BDNF/TrkB activation; mitochondrial protection; caspase inhibition
Necroptosis	RIPK1/RIPK3/MLKL activation; DAMP release	Neurons/glial cells; hippocampus	Membrane rupture, microglial activation, amplification of neuroinflammation	RIPK1/RIPK3 or MLKL inhibition
Pyroptosis	NLRP3//caspase-1/GSDMD; IL-1β/IL-18	Microglia, astrocytes; hippocampus	Neuroinflammation, glial dysfunction, impaired neurogenesis and synaptic function	NLRP3, caspase-1 and GSDMD inhibition
Ferroptosis	System Xc^−^/GSH/GPX4 dysfunction; iron overload; lipid peroxidation; NCOA4-mediated ferritinophagy	Mainly neurons; hippocampus and other mood-related regions	Oxidative injury, lipid membrane damage, synaptic dysfunction	GPX4 restoration; iron chelation; ferroptosis inhibitors

## 2 The role and mechanisms of apoptosis in depression

### 2.1 Activation characteristics of apoptotic pathways

During the pathophysiological progression of depression, aberrant activation of apoptosis represents a critical event contributing to neuronal loss in key brain regions involved in emotional regulation (Zhang Y. et al., 2024). Evidence from postmortem brain tissues of patients with depression and chronic stress-induced animal models consistently demonstrates increased apoptotic activity in vulnerable regions, particularly the hippocampal CA1 region and the prefrontal cortex (Zhang Y. et al., 2026; Zhang L. et al., 2024). These alterations include increased cleaved caspase-3 expression and an elevated ratio of the pro-apoptotic protein Bax to the anti-apoptotic protein Bcl-2 (Shelton et al., 2011). Among the apoptotic pathways implicated in depression, the intrinsic mitochondrial pathway is considered the predominant mechanism. Chronic exposure to corticosterone or chronic unpredictable mild stress (CUMS) induces mitochondrial outer membrane permeabilization (MOMP), resulting in the release of cytochrome c from mitochondria into the cytoplasm (Wu et al., 2023; Wang et al., 2025; Ciubuc-Batcu et al., 2024). Cytochrome *c* subsequently binds apoptotic protease-activating factor 1 (Apaf-1) and promotes apoptosome assembly. The apoptosome recruits and activates procaspase-9, which subsequently activates executioner caspases, particularly caspase-3 and caspase-7 (Scaini et al., 2022; Jiang and Wang, 2000; Holper et al., 2019). This event subsequently triggers the downstream caspase cascade, ultimately leading to programmed neuronal death (Wu et al., 2023). Besides, the extrinsic death receptor-mediated apoptotic pathway also contributes to depression-associated neuronal loss (Xiong et al., 2025). Under depressive conditions, increased levels of inflammatory mediators, including TNF-α and Fas ligand (FasL), activate caspase-8-dependent apoptotic signaling, thereby further exacerbating neuronal injury (Xiong et al., 2025). TNFR1 signaling can recruit TRADD, FADD, RIPK1, and procaspase-8 to form a cytosolic death-inducing complex, whereas Fas directly recruits FADD and procaspase-8 to assemble the death-inducing signaling complex (Gao et al., 2024; Zheng et al., 2025; Erkilinc et al., 2025).

### 2.2 Apoptotic regulatory factors and depression pathogenesis

The execution of apoptosis in depression is governed by a constellation of regulatory intermediates that transduce upstream stress signals into downstream cell death programs. Central to this network is brain-derived neurotrophic factor (BDNF) signaling. BDNF deficiency impairs TrkB-mediated PI3K/Akt survival cascades, thereby lowering the apoptotic threshold and priming neurons for death initiation (Hartig and Nemeş, 2023). The cellular stress sensor p53 is markedly upregulated in depressive paradigms. Activated p53 transcriptionally upregulates pro-apoptotic effectors (Bax) while repressing anti-apoptotic counterparts (Bcl-2), thereby directly potentiating mitochondrial apoptotic pathway activation (Wu et al., 2023). Furthermore, glucocorticoid receptor (GR)-mediated apoptotic signaling is critically involved in stress-induced neuronal damage. In CUMS model, GR translocation into mitochondria promotes mitochondrial dysfunction and apoptosis under chronic stress conditions (Wu et al., 2023; Ciubuc-Batcu et al., 2024; Wang B. et al., 2023). The glucocorticoid receptor co-chaperone FKBP5 also contributes to depression-associated apoptotic regulation. Altered FKBP5 expression and DNA methylation patterns have been linked to hypothalamic structural changes, suggesting a potential molecular connection between hypothalamic–pituitary–adrenal axis dysregulation and neuronal apoptosis (Suh et al., 2021). Together, these apoptosis-related regulators form a complex molecular network that integrates stress responses, neurotrophic deficits, endocrine dysfunction, and neuronal vulnerability in depression.

## 3 The roles of necroptosis and pyroptosis in depression

### 3.1 Mechanisms of necroptosis in depression

Necroptosis, a caspase-independent form of regulated necrosis, is executed primarily through the canonical RIPK1/RIPK3/MLKL signaling axis. Unlike apoptosis, which is characterized by caspase-dependent and immunologically silent cell elimination, necroptosis induces membrane rupture and the release of intracellular damage-associated molecular patterns (DAMPs), thereby triggering robust inflammatory responses (Zheng et al., 2025; Yang et al., 2025; Serna-Rodríguez et al., 2022; Hu et al., 2024). The aberrant activation of necroptosis contributes to the pathogenesis of depression. In a CUMS-induced mouse model of depression, hippocampal expression levels of RIPK3 and phosphorylated MLKL (p-MLKL), two key molecular markers of necroptosis activation, were significantly elevated (Zeb et al., 2022; Cao et al., 2021; Yang et al., 2025). Moreover, increased necroptotic signaling was positively correlated with behavioral despair phenotypes, including prolonged immobility time in the tail suspension test (Yan et al., 2021). Consistently, enhanced MLKL phosphorylation has also been observed in lipopolysaccharide (LPS)-induced inflammatory depression models, further supporting the involvement of necroptosis in inflammation-associated depressive pathology (Zheng et al., 2025). TNF-α represents a critical molecular bridge linking inflammatory activation and programmed cell death in depression (Zheng et al., 2025). Under physiological conditions, TNF-α signaling through TNFR1 can induce either apoptosis or survival pathways depending on cellular context. However, under chronic inflammatory conditions, impaired caspase-8 activation or inhibition of caspase-8-mediated cleavage can redirect cell fate from apoptosis toward necroptosis by allowing RIPK1/RIPK3 complex formation and subsequent MLKL activation (Zhang et al., 2020). This transition promotes a highly inflammatory form of cell death. Necroptotic cells release abundant DAMPs, including HMGB1, extracellular ATP, and mitochondrial components, which activate surrounding microglia and amplify neuroinflammatory responses (Zheng et al., 2025; Qiu et al., 2026). This creates a self-perpetuating “necroptosis–inflammation–secondary cell death” positive feedback loop, thereby accelerating neuronal dysfunction and progressive neurodegenerative changes associated with depression (Guo et al., 2025).

### 3.2 Pyroptosis in amplifying neuroinflammation

Pyroptosis is a highly inflammatory form of programmed cell death driven by inflammatory caspases, including caspase-1, caspase-4/5 in humans, and caspase-11 in mice (Li et al., 2021b; Zhao et al., 2026; Wan et al., 2022). Unlike apoptosis, pyroptosis is characterized by gasdermin-mediated membrane pore formation and massive release of pro-inflammatory cytokines, making it a key mechanism linking cellular stress to immune activation (Wang et al., 2026). In depression, pyroptotic activation is closely associated with abnormal activation of the NLRP3 inflammasome. Clinical studies have demonstrated that patients with major depressive disorder exhibit elevated activation of the NLRP3 inflammasome in peripheral blood and cerebrospinal fluid, accompanied by increased expression of inflammasome-associated components and downstream inflammatory cytokines compared with healthy individuals (Deng et al., 2022; Kouba et al., 2022). The NLRP3 inflammasome senses diverse danger signals, including extracellular ATP, reactive oxygen species (ROS), mitochondrial dysfunction, and mitochondrial DNA leakage (Munshi et al., 2025; Newman and Shadel, 2023). Upon activation, NLRP3 recruits the adaptor protein ASC, leading to the cleavage of pro-caspase-1 into active caspase-1 (Garrosa-Jiménez et al., 2021). Activated caspase-1 subsequently cleaves GSDMD to generate the N-terminal fragment GSDMD-N, which oligomerizes within the plasma membrane to form pores and induce pyroptotic lysis. Caspase-1 also promotes the maturation and secretion of IL-1β and IL-18, further amplifying inflammatory signaling (Zhou et al., 2026). In depressive pathology, pyroptosis of hippocampal microglia has emerged as a central mechanism driving neuroinflammation. IL-1β released from pyroptotic microglia directly impairs synaptic function and suppresses hippocampal neurogenesis, ultimately contributing to cognitive impairment and emotional dysregulation (Shen et al., 2026). Furthermore, astrocyte pyroptosis has been identified as an important contributor to astrocytic loss in depressive animal models. Genetic inhibition of GSDMD, caspase-1, as well as astrocytic NLRP3 inflammasome activation reduces depressive-like behaviors and suppresses pyroptosis-associated molecular alterations, highlighting the essential role of glial cell pyroptosis in depression pathogenesis (Li et al., 2021a; McColgan et al., 2026). Therefore, glial pyroptosis as an important cellular mechanism linking inflammatory stress to neuronal dysfunction in depression.

### 3.3 Crosstalk between necroptosis and pyroptosis in depression

Necroptosis and pyroptosis should not be considered independent cell death pathways; rather, they form an interconnected regulatory network that integrates inflammatory signaling and cellular fate decisions (Zheng et al., 2025; Zhang et al., 2025). Recent evidence has revealed direct molecular interactions between these pathways. RIPK3, traditionally recognized as a core regulator of necroptosis, can directly promote NLRP3 inflammasome activation through phosphorylation-dependent mechanisms, establishing a molecular convergence point between necroptotic signaling and inflammasome-mediated pyroptosis (Zhao et al., 2024; Doglio et al., 2023). This interaction may be particularly relevant in depression, where chronic stress and persistent inflammation create a permissive environment for multiple forms of inflammatory cell death. During pyroptotic activation, IL-1β released from inflammasome-activated cells can stimulate TNFR1 signaling and enhance RIPK1 expression, thereby lowering the threshold for necroptotic activation and facilitating the transition toward highly inflammatory cell death programs (Zhang et al., 2020). The reciprocal amplification between necroptosis and pyroptosis provides a potential explanation for the persistent neuroinflammatory state observed in chronic depression. Accordingly, simultaneous targeting of these pathways may provide superior therapeutic efficacy compared with inhibition of either pathway alone (Feng et al., 2025; Hou et al., 2025). Experimental studies have demonstrated that combined administration of a RIPK3 inhibitor and an NLRP3 inhibitor produces stronger antidepressant-like effects than either intervention alone by simultaneously reducing necroptosis-associated DAMP release and pyroptosis-associated IL-1β production (Zhao et al., 2024). Therefore, multi-target strategies targeting interconnected inflammatory cell death pathways may represent a promising therapeutic approach for treatment-resistant depression. By disrupting the vicious cycle of cell death–inflammation amplification, such approaches may provide broader neuroprotective effects and facilitate the development of next-generation antidepressant therapies (Jia et al., 2026; Zhang G. et al., 2026).

## 4 Emerging role of ferroptosis in the pathogenesis of depression

### 4.1 Ferroptosis and abnormal iron metabolism in depression-susceptible brain regions

Ferroptosis is a newly recognized form of regulated cell death characterized by iron-dependent lipid peroxidation and distinct molecular features, including depletion of intracellular glutathione (GSH) and impaired activity of GPX4 (Li and Huang, 2022). Increasing evidence suggests that ferroptosis may represent an important mechanism underlying neuronal vulnerability in depression. Neuroimaging studies in patients with depression have revealed abnormal iron accumulation in brain regions associated with mood regulation, including the hippocampus and basal ganglia, providing structural evidence supporting the involvement of iron dysregulation in depressive pathology (Duan et al., 2022). Under depressive conditions, neuronal iron homeostasis is disrupted, characterized by increased expression of transferrin receptor 1 (TfR1), which facilitates cellular iron uptake, accompanied by reduced expression of the iron exporter ferroportin, resulting in excessive intracellular iron accumulation (Zhu et al., 2025). This iron overload provides abundant substrates for Fenton chemistry, in which ferrous iron (Fe^2+^) catalyzes the conversion of hydrogen peroxide into highly reactive hydroxyl radicals, thereby inducing severe oxidative stress (Zhang C. et al., 2026; Zhang J. et al., 2026). In addition to iron dysregulation, alterations in membrane lipid composition further increase neuronal susceptibility to ferroptosis. Polyunsaturated fatty acids (PUFAs), particularly ferroptosis-sensitive phospholipids containing polyunsaturated fatty acyl chains, are enriched in neuronal membranes of individuals with depression (Sublette et al., 2024). Due to their high content of double bonds, these lipids are particularly vulnerable to free radical-mediated oxidation, thereby increasing the availability of substrates for lipid peroxidation (Jacquemyn et al., 2024). Therefore, the combination of disrupted iron metabolism and altered membrane lipid composition establishes a ferroptosis-prone environment in depression-associated brain regions. These alterations compromise neuronal antioxidant capacity and increase vulnerability to oxidative damage, ultimately contributing to neuronal dysfunction and impaired emotional regulation.

### 4.2 Regulatory pathways of ferroptosis in depression

The GPX4/GSH axis represents the central defense system against ferroptotic cell death. Chronic stress exposure suppresses the activity of system Xc^−^, a cystine/glutamate antiporter responsible for cystine uptake, resulting in reduced cystine availability and impaired GSH synthesis (Zhang C. et al., 2026; Li et al., 2023). Consequently, diminished GSH levels compromise GPX4 activity, leading to the accumulation of toxic lipid peroxides and increased neuronal susceptibility to ferroptosis (Costa et al., 2023). Beyond the canonical GPX4-dependent pathway, ferroptosis suppressor protein 1 (FSP1) functions as an alternative antioxidant defense mechanism by maintaining coenzyme Q10 (CoQ10)-dependent suppression of lipid peroxidation. Recent studies suggest that reduced FSP1 expression in depressive models may further weaken ferroptosis resistance and promote neuronal damage (Li N. et al., 2022). Autophagy-dependent regulation of iron metabolism also contributes to ferroptotic vulnerability. NCOA4-mediated ferritinophagy selectively degrades ferritin and releases large amounts of labile iron, thereby amplifying iron-dependent lipid peroxidation and facilitating ferroptotic cell death under stress conditions (Li C. et al., 2021). Importantly, ferroptosis is closely interconnected with neuroinflammation, forming a self-amplifying pathological feedback loop. Membrane phospholipid composition represents another critical determinant of ferroptotic susceptibility. ACSL4 activates polyunsaturated fatty acids, particularly arachidonic and adrenic acids, by converting them into acyl-CoA derivatives (Chen et al., 2023; Liu G. et al., 2025; Magarditchian et al., 2026). LPCAT3 subsequently incorporates these fatty acyl chains into membrane phospholipids (Zhang et al., 2021; Liu X. et al., 2025). Besides, PUFA-containing phospholipids provide readily oxidizable substrates for enzymatic and nonenzymatic lipid peroxidation (Liu G. et al., 2025; Wang L. et al., 2023). Accordingly, ACSL4/LPCAT3 activity will establish a ferroptosis-prone membrane lipid profile, whereas suppression of this remodeling pathway may reduce neuronal sensitivity to ferroptotic stress (Liu G. et al., 2025; Huang et al., 2024; Cui et al., 2023). Under oxidative stress, NRF2 escapes KEAP1-mediated degradation, translocates into the nucleus, and induces genes involved in cystine uptake, GSH synthesis, lipid-peroxide detoxification, NADPH regeneration, and iron homeostasis, including *SLC7A11*, *GCLC*, *GCLM*, *GPX4*, *NQO1*, and *HMOX1* (Dang et al., 2022; Wang et al., 2024; Zuo et al., 2022). Impaired NRF2 signaling in depression-related conditions may therefore weaken multiple antioxidant systems and facilitate iron-dependent lipid peroxidation (Zuo et al., 2022). Lipid peroxidation products, such as 4-hydroxynonenal (4-HNE), can activate NF-κB signaling and promote the production of pro-inflammatory cytokines, including TNF-α and IL-6 (Chen J. et al., 2024). Conversely, inflammatory cytokines can further disrupt iron homeostasis by enhancing TfR1 expression and promoting intracellular iron accumulation, thereby reinforcing ferroptotic processes. This bidirectional interaction between oxidative damage and inflammation creates a vicious cycle that accelerates neuronal injury in depression.

### 4.3 Therapeutic potential of ferroptosis inhibitors in depression models

Pharmacological targeting of ferroptotic pathways has attracted increasing attention as a potential therapeutic strategy. The iron chelator deferoxamine (DFO) has demonstrated antidepressant-like effects in LPS-induced depressive models by reducing hippocampal iron accumulation, decreasing lipid peroxidation products such as malondialdehyde (MDA), and alleviating behavioral abnormalities including anhedonia and reduced locomotor activity (Zhu et al., 2025). Lipophilic radical-trapping antioxidant Liproxstatin-1, a potent ferroptosis inhibitor, has shown significant neuroprotective effects in CUMS models. Treatment with Liproxstatin-1 restores hippocampal GPX4 activity, preserves the expression of postsynaptic density protein 95 (PSD-95), and reverses stress-induced depressive-like behaviors, indicating that ferroptosis inhibition can effectively restore neuronal function and synaptic integrity (Zhu et al., 2025). Furthermore, emerging evidence suggests that combining ferroptosis-targeting agents with conventional antidepressants may provide synergistic therapeutic benefits. For example, ferroptosis inhibition combined with selective serotonin reuptake inhibitors (SSRIs), such as sertraline, produces enhanced antidepressant effects compared with either intervention alone, suggesting that ferroptotic signaling may represent an independent therapeutic target in depression (Wang L. et al., 2023). This combined therapeutic strategy may be particularly beneficial for depression subtypes characterized by elevated neuroinflammation and oxidative stress, as ferroptosis inhibitors can not only directly suppress lipid peroxidation but also attenuate inflammatory responses. Thus, ferroptosis inhibition represents a promising component of mechanism-based combination therapy for depression (Figure 1).

**FIGURE 1:**
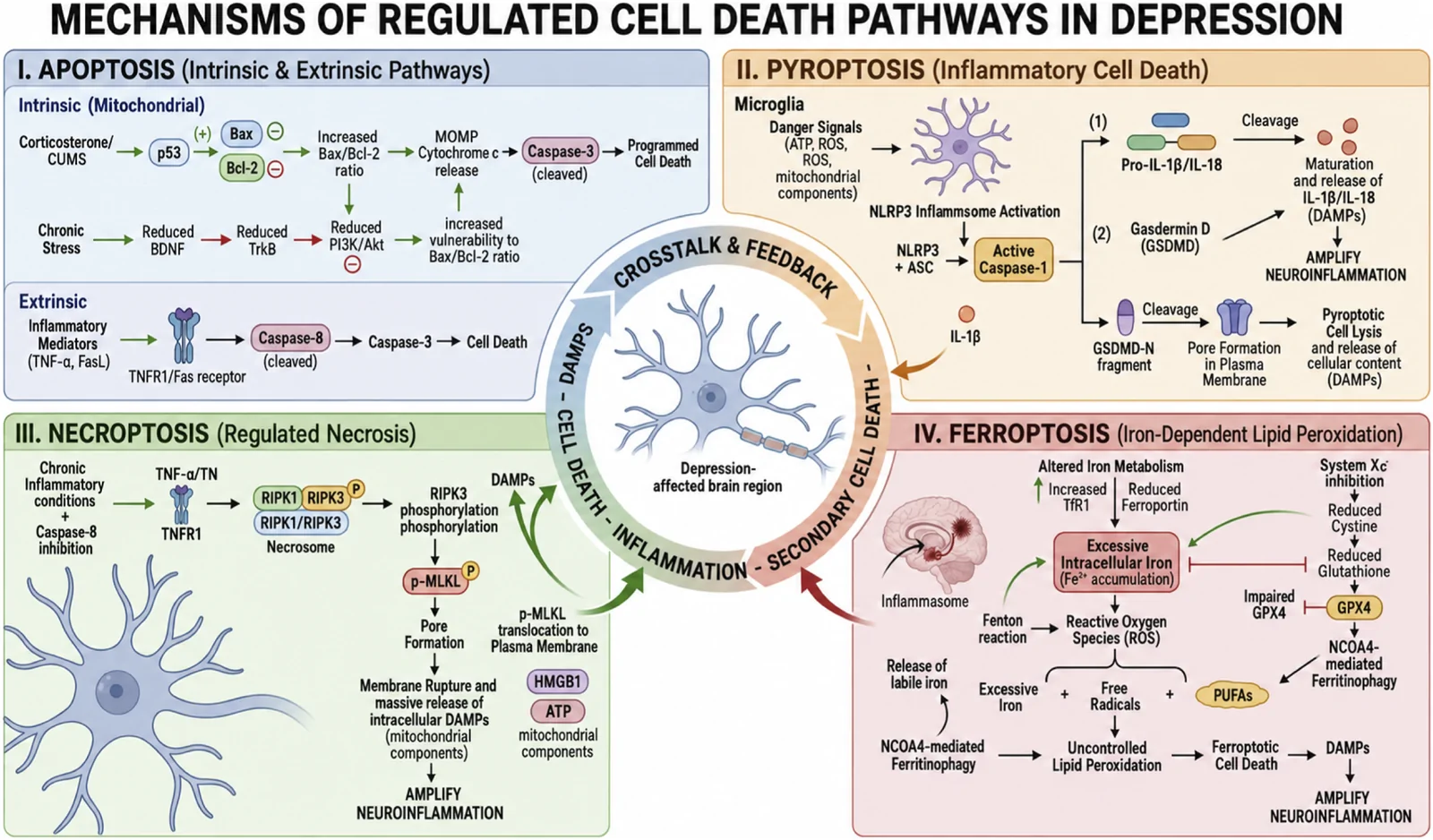
Mechanisms of regulated cell death pathways in depression.

## 5 Interconnected cell death networks in depression

Cell-type specificity shapes RCD in depression. Neurons, with high metabolic demands, are particularly susceptible to apoptosis and ferroptosis. Hippocampal tissues in depression and stress models exhibit ferroptosis-consistent changes, including reduced GSH, increased MDA, and altered FTO and BECN1 expression (Meng et al., 2025; Zhang C. et al., 2026; Zhang et al., 2023). TNFSF10 is increased in patients with depression and comorbid type 2 diabetes and linked to cognitive decline, supporting neuronal apoptotic involvement (Perfetto et al., 2026). Microglia respond to inflammatory stress through pyroptosis; 3-hydroxykynurenine induces mitochondrial-dependent pyroptosis and IL-1β release, and microglial cytokines may promote neuronal apoptosis or necroptosis (Shen et al., 2026). Astrocytes maintain metabolic and trophic support but undergo NLRP3/caspase-1/GSDMD-dependent pyroptosis under chronic stress. Inhibiting this pathway reduces astrocytic loss and depressive-like behaviors (Li et al., 2021a; Li S. et al., 2022; Han et al., 2025; Xia et al., 2023). RCD is dynamic across disease stages and brain regions. Early stress may activate apoptosis, whereas chronic inflammation increasingly engages necroptosis and pyroptosis. Programmed cell-death signatures change with disease progression and correlate with immune activation (Xiong et al., 2025). Pathological signals may spread from hippocampal regions to CA3–CA1 circuits and PFC neurons. ENIGMA analyses identified reduced hippocampal CA1 thickness and surface area and altered basolateral amygdala structure, particularly in recurrent depression (Ho et al., 2022). Volumetric differences among amygdalar subregions further indicate heterogeneity (Roddy et al., 2021). Such changes may contribute to persistent circuit dysfunction and symptoms. Thus, RCD pathways may mark distinct disease stages and regional patterns, supporting stage- and region-specific interventions. RCD pathways share upstream drivers, including oxidative stress, mitochondrial dysfunction, and inflammatory signaling, single-pathway inhibition may be inadequate (Scaini et al., 2022; Grotle et al., 2022). Integrated strategies could combine caspase, RIPK1/RIPK3, NLRP3, and GPX4-directed interventions. Pyroptosis-related gene signatures distinguish depression and define immunologically distinct subtypes (Deng et al., 2022; Li et al., 2021a). Single-cell and spatial transcriptomics may localize abnormalities, including CDKN1A and RHOB dysregulation (Liu et al., 2026). In addition, Ginsenoside Rb1 exemplifies convergent targeting by modulating FTO, BECN1, and m6A methylation to suppress ferroptosis and apoptosis (Meng et al., 2025; Jiang et al., 2022; Lin et al., 2024). Overall, these findings support integrated, stage-specific, and cell-type-informed approaches for precision treatment of depression.

## 6 Conclusion

Depression is increasingly understood as a multifactorial neurobiological disorder in which chronic stress, neuroinflammation, oxidative imbalance, mitochondrial dysfunction, and impaired neurotrophic support converge to disrupt neuronal homeostasis. Within this framework, regulated cell death provides a mechanistic bridge between molecular stress and neural injury. Apoptosis, mediated predominantly through mitochondrial and death-receptor pathways, may contribute to neuronal loss in vulnerable regions, whereas necroptosis and pyroptosis amplify tissue inflammation through membrane rupture, DAMP release, inflammasome activation, and cytokine production. Ferroptosis adds a distinct iron- and lipid-peroxidation-dependent mechanism that is closely linked to impaired GPX4/GSH, FSP1, NRF2, and autophagy-associated defenses. These pathways are not independent; rather, they interact dynamically across neurons, microglia, and astrocytes and may evolve according to disease stage, brain region, and inflammatory context.

This integrated model has therapeutic implications. Targeting individual pathways with caspase, RIPK1/RIPK3, NLRP3, iron-chelating, or lipid-peroxidation inhibitors may provide neuroprotection, but crosstalk among RCD programs suggests that single-target approaches may be insufficient for heterogeneous or treatment-resistant depression. Future therapies should prioritize shared regulatory nodes, including mitochondrial dysfunction, redox imbalance, inflammasome activation, and defective autophagy, while incorporating conventional antidepressants. Single-cell and spatial multi-omics, together with advanced human brain models, may identify cell-type-specific vulnerabilities and molecular subtypes. However, pathway-associated markers alone cannot establish execution of a particular death program; rigorous studies should integrate biochemical, morphological, functional, and pharmacological evidence. Defining the temporal and spatial sequence of RCD activation will be essential for developing stage-specific interventions and translating these mechanisms into precise, mechanism-based treatments for depression across diverse patient populations.
